# Designing Ruby: Protocol for a 2-Arm, Brief, Digital Randomized Controlled Trial for Internalized Weight Bias

**DOI:** 10.2196/31307

**Published:** 2021-11-25

**Authors:** Christina M Hopkins, Hailey N Miller, Taylor L Brooks, Lihua Mo-Hunter, Dori M Steinberg, Gary G Bennett

**Affiliations:** 1 Duke Global Digital Health Science Center Duke University Durham, NC United States; 2 School of Nursing Duke University Durham, NC United States; 3 Equip Health San Diego, CA United States

**Keywords:** obesity, stigma, mHealth, mindfulness, self-compassion, mobile phone

## Abstract

**Background:**

Weight bias internalization, also known as *weight self-stigma*, is a serious health concern for individuals with higher body weight. Weight bias internalization is associated with the greater avoidance of health care and health-promoting activities, disordered eating, social isolation, and weight gain. Elevated weight bias internalization has been associated with low self-compassion, yet few investigations have explored self-compassion as a potential mechanism for reducing internalized weight bias.

**Objective:**

Ruby is a 2-arm randomized controlled trial that was designed to test the efficacy of a 4-week digital self-compassion intervention to reduce internalized weight bias compared with a wait-list control.

**Methods:**

Adults with elevated internalized weight bias and a BMI of >30 kg/m^2^ (N=80) were recruited. Ruby is a standalone digital trial that will be delivered entirely via a smartphone and will involve web-based data collection and text messages. The intervention content will include psychoeducation and daily mindfulness practices with a focus on self-compassion and body concerns. We will use intent-to-treat analyses to examine changes in weight bias internalization throughout time by treatment arm. The analyses will be conducted by using one-way analysis of covariance models and linear mixed models.

**Results:**

The protocol was designed in May 2020 and approved in December 2020. Data collection is currently underway.

**Conclusions:**

Ruby will be the first digital standalone, self-compassion–based intervention designed to reduce internalized weight bias. Owing to its standalone digital delivery, Ruby may be a highly scalable treatment for internalized weight bias that can be delivered on its own or combined with other treatments. We expect Ruby to be accessible to many, as participants can access the digital intervention at times of the day that are the most convenient in their schedule and are not burdened by in-person time commitments, which can be a barrier for participants with competing demands on their time and resources. If efficacious, Ruby will be poised to expand a burgeoning body of literature related to psychological intervention in this area.

**Trial Registration:**

ClinicalTrials.gov NCT04678973; https://clinicaltrials.gov/ct2/show/NCT04678973

**International Registered Report Identifier (IRRID):**

DERR1-10.2196/31307

## Introduction

The physiological impact of obesity is often seen as the primary risk factor associated with obesity, but the *psychosocial* burden of obesity is harmful and may pose a greater risk. Weight stigma—the persistent devaluation, stereotyping, or discrimination of a person based on weight—is of paramount concern and a leading risk factor for adverse health outcomes [1].

Weight stigma is pervasive; experiences of weight stigma have been observed as early as at the age of 3 years [2]. Estimates of weight stigma experiences (ie, teasing, unfair treatment, or discrimination) across all demographics range from 20% to 40%. Rates tend to be higher in younger adults than older adults, women, individuals who self-identify as White, and people in higher weight classes [1]. Despite the myriad health consequences of weight stigma, these attitudes are commonly accepted in society because of the belief that body shame will result in motivation to lose weight [3]. However, evidence indicates that the opposite is true: experience of weight stigma is associated with weight gain, social isolation, increased binge eating episodes, and avoidance of health care services and physical activity [4].

Weight stigma (ie, social rejection because of weight) emanates from weight bias (ie, social prejudice regarding weight). Weight bias can also be internalized; that is, individuals can apply these societal beliefs about being overweight to themselves, begin to believe these stereotypes themselves, and self-stigmatize. This internalized weight bias (sometimes referred to as *weight self-stigma*) results in numerous serious health consequences, over and above those that may be associated with simply experiencing weight stigma [5].

In adults, internalized weight bias has been shown to result in an increased risk of metabolic syndrome, increased risk of eating disorder development, elevated triglycerides, and decreased quality of life after controlling for BMI [4,6]. The stress of internalized weight bias is often compared with other chronic discriminatory stressors (eg, racism). Dysregulation of the hypothalamic-pituitary-adrenal axis and consequent long-term elevation in cortisol levels have been observed in individuals who internalize weight bias, replicating findings in investigations of caregiver burden [7], employee burnout [8], childhood bullying [9], and persistent racism [10].

Internalized weight bias is associated with increased rates of maladaptive eating patterns and avoidance of healthy behaviors, such as physical activity [11,12]. Individuals with internalized weight bias are also less likely to attend preventive care visits to their physician, less likely to complete health screenings in accordance with guidelines, and more likely to switch health care providers [4]. This three-pronged issue—maladaptive eating patterns, physical inactivity, and fewer physician visits—may contribute to the poor health and well-being of those with internalized weight bias.

Recent evidence demonstrates that adaptive coping responses mediate the association between experiencing weight stigma and negative health outcomes [12]. Himmelstein et al [12] found that health outcomes were directly associated with how individuals responded to experiences of weight stigma. For instance, experiencing weight stigma was associated with greater depressive symptoms, but this effect was mediated by coping with negative affect. Furthermore, weight stigma was indirectly associated with lower depressive symptoms through the mediating effect of coping via healthy lifestyle behaviors such as eating healthy foods and exercising. This suggests that the consequences of weight stigma might be more readily attributed to an individual’s response to stigma than to stigma alone. If internalization of weight bias is conceptualized as a response to experiences of weight stigma (in the way that Himmelstein et al [12] conceptualize negative affect), it becomes clear that intervention on the internalization of weight bias is possible and essential to improve health.

Acceptance- and mindfulness-based psychotherapies are well-positioned to disrupt the pathway from weight stigma to its negative health consequences. Recent investigations have begun to test the effects of acceptance- and mindfulness-based psychotherapies on weight stigma and related constructs (eg, body dissatisfaction), resulting in positive effects on reducing self-directed stigma [13]. Mindfulness skills are also known to increase several aspects of metacognition, including decentering from thoughts and emotions and reperceiving negative experiences, which could significantly impact an individual’s response to experiences of weight stigma and thus, the development of internalized weight bias.

One such mindfulness skill is self-compassion. Self-compassion is a multidimensional mindfulness-based construct consisting of 3 parts: self-directed kindness, a sense of common humanity, and mindfulness [14]. Recent investigations have highlighted self-compassion as an important treatment target in patients with internalized weight bias and related concerns. Self-compassion is associated with greater overall psychological health [15], and a more recent systematic review suggests that amplifying self-compassion can ameliorate body image disturbance and eating pathology [16]. Both experienced and internalized weight bias have been linked to low self-compassion and low self-kindness in young adults [17] and adults [11]. In a clinical trial, self-compassion intervention was associated with greater reductions in body dissatisfaction than in wait-list control [18]. Overall, these findings suggest that self-compassion may improve well-being and reduce internalized weight bias. To date, no intervention has tested the efficacy of a brief self-compassion intervention on internalized weight bias. We aim to fill this gap by developing and testing a digitally delivered self-compassion mindfulness intervention called Ruby.

## Methods

### Overview

Ruby is a 2-arm, 4-week randomized controlled trial (N=80) testing the efficacy of a digital mindfulness-based self-compassion intervention for internalized weight bias compared with a wait-list control. The primary outcome is a 4-week change in self-reported weight bias internalization, as measured by the Weight Bias Internalization Scale [5]. Secondary outcomes include 4-week changes in self-compassion, body appreciation, cognitive flexibility related to weight, and other related constructs. The Duke University institutional review board approved this protocol in December 2020.

### Population

#### Overview

Ruby will enroll adults with obesity and internalized weight bias. Eligible participants will be at least 18 years old, report a BMI of at least 30 kg/m^2^, have experienced weight bias, report elevated internalized weight bias as determined by a score of at least 4.0 on the Weight Bias Internalization Scale, have a smartphone, be willing to receive multiple text messages per day and engage in mindfulness practice for up to 20 minutes per day, live in the United States EST zone, and be able to read and write in English fluently. A Weight Bias Internalization Scale cutoff score of 4.0 has been used in prior research to determine *high* internalization of weight bias [19]. Reports on population norms of this measure indicate a cutoff score of 4.0 will capture women in the 80th percentile and men in the 90th percentile of internalized weight bias [20]. Participants will be excluded if they do not meet the eligibility criteria listed above, if they are already regular meditators (ie, meditate for at least 1 day per week for more than 1 week), are currently engaged in other treatments similar to Ruby (ie, in a mindfulness program, working on weight-related distress, or actively trying to lose weight), or have recently undergone bariatric surgery.

#### Power and Sample Size

We will recruit 80 adults aged >18 years who have English language proficiency, a BMI higher than 30 kg/m^2^, and report weight bias internalization equal to or greater than a score of 4.0. The sample size was calculated based on the primary outcome using G*Power with a medium effect size determined based on the literature (effect size=0.5; power=0.95). We predict a 1-point reduction in the Weight Bias Internalization Scale score, based on prior literature in this field. We will need a minimum of 55 participants to be adequately powered; we estimate a retention rate of 70% and thus inflated sample size to account for attrition for a final sample size of 80 participants.

### Recruitment

The target enrollment for this trial is 80 participants, with 40 participants in each group. Participants will be recruited from anywhere within the EST zone in the United States to ensure consistent timing of text messages across participants while maximizing reach. To recruit eligible participants, we will use a multipronged web-based approach. We will list information about Ruby on a clinical trial registry from the National Institutes of Health and use social media platforms (ie, Instagram, Twitter, and Facebook) and professional networks to distribute recruitment materials. We will also use ResearchMatch, a national health volunteer registry, to identify potentially eligible participants and notify them about Ruby. Of note, 75% of ResearchMatch registrants are White individuals, 70% are female, and 90% are non-Hispanic or Latino individuals. All recruitment sources will direct interested participants to the study website to provide details about the intervention, eligibility criteria, and contact information for study staff should they have questions about Ruby before continuing to the remaining screening procedures.

### Screening, Consent, and Baseline Assessments

The prescreening eligibility survey will be administered through the REDCap (Research Electronic Data Capture) website (Vanderbilt University), a secure web-based software platform designed for data collection and management in research studies. The survey will collect participant contact information, recruitment sources, and questions to determine eligibility based on the criteria listed above. The participant will be automatically notified of their eligibility status and, if eligible, will be directed to the web-based consent form. If ineligible, they will be directed to nationally available resources for body image concerns, mental health resources, and reading materials that they might find supportive.

Eligible participants will proceed to the informed consent document hosted on the REDCap platform. Participants will be encouraged to read the informed consent document carefully and contact study staff via email if they have any questions or hesitations about participating in Ruby. If they are interested and agree to the study procedures, they will provide their electronic signatures to indicate their informed consent.

Participants who provide informed consent will be automatically emailed a unique link to their baseline surveys and instructed to complete them within 24 hours. The baseline surveys will include several instruments to measure baseline values on a variety of constructs, as described in more detail below. These surveys can be completed in a web browser using smartphones or personal PCs. Once participants complete their final baseline assessment survey, REDCap will automatically send an email to a study staff member to indicate that a new participant is ready for randomization.

### Randomization

**Figure 1:**
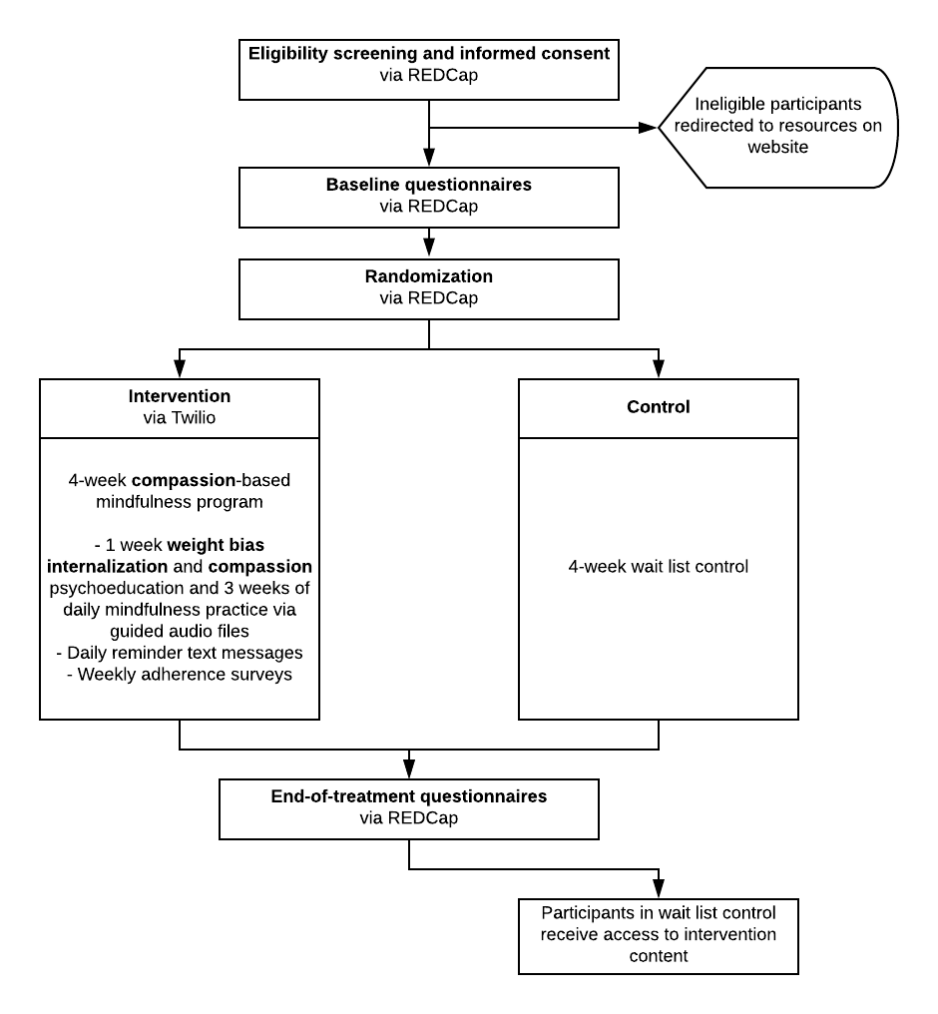
Study flow. REDCap: Research Electronic Data Capture.

Participants who are eligible, provide informed consent, and complete baseline questionnaires will be randomized to one of two trial arms: intervention or wait-list control. Randomization will be conducted using simple random sampling; a randomization allocation table will be created using Microsoft Excel and uploaded to REDCap. Once the study staff is notified of a new participant eligible to be randomized, they will complete a random assignment using the randomization module in REDCap, which automatically assigns the participant to 1 of 2 groups and locks their randomization data fields. Study staff will not be blinded to randomization; however, this is not expected to impact trial outcomes as both treatment arms are completely preprogrammed, and thus, the potential bias of study staff could not impact intervention delivery. Randomization in REDCap will automatically trigger the delivery of text messages to provide notifications to the participant of their assigned group and orientation about what to expect. Figure 1 shows a summary of the study flow.

### Intervention Delivery

As described above, Ruby will be delivered as a standalone digital health intervention via a smartphone. All data collection, similar to screening and baseline assessments, will be automated and completed via a web browser. Intervention content is delivered via text messages with links to additional intervention content. Given the known nonuse attrition common in mobile apps, text messages provide an excellent delivery method for Ruby [21]. Unlike a mobile app, text messages cannot be uninstalled by the user nor can they be offloaded by the mobile operating system because of the lack of available storage on the smartphone. Although the user can go to additional lengths to block text messages, we presume this will only occur for a small subset of Ruby participants. Ruby is preprogrammed and automated and thus requires no synchronous human contact from a clinician or study team member.

### Treatment Arms

#### Wait-list Control Group

Immediately following randomization, participants randomized to the control group will receive 2 brief text messages notifying them of their group assignment and extending gratitude for their patience. The control group participants will receive one text message halfway through their waiting period (ie, on day 14) and one text message at the end of their waiting period (ie, on day 28). The text on day 14 will notify them of the halfway point, and the text on day 28 will ask them to complete their end-of-treatment surveys. No additional intervention content or resources will be provided to the control group participants during the trial period. After completion of their end-of-treatment surveys, participants will be entered into a raffle to win study compensation and will be offered access to the Ruby intervention. If they opt in, they will begin receiving the intervention the next day.

#### Intervention Group

**Figure 2:**
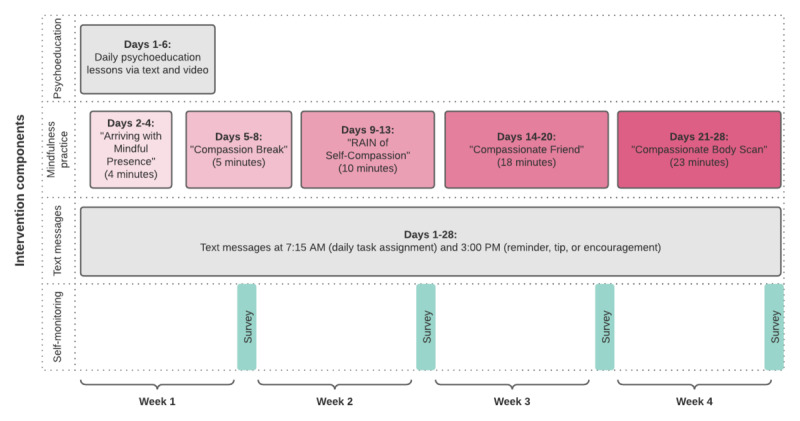
Overview of the intervention components and delivery schedule.

Immediately following randomization, participants randomized to the intervention group will be sent a series of text messages notifying them of their group assignment. They will also receive a link to a Ruby orientation page on the study website. The orientation page will outline participation expectations and study details, including when text messages will be sent and from what phone number, what participants’ daily time commitment will be, and how to get in touch with study staff if they need technical assistance. An outline of the intervention activities each day has been provided in Figure 2; the duration of each mindfulness recording is provided.

#### Psychoeducation

Participants in the intervention group will receive a text message from day 1 to day 6 of the intervention that includes a link to psychoeducation material (Figure 2). These materials will be hosted on a hidden page on the study website. The purpose of the psychoeducation material is to orient users to their upcoming experience, provide education about internalized weight bias, the nervous system, and weight bias as a chronic stressor, the utility of self-compassion practice to regulate physiological and psychological targets, and guidance on establishing and maintaining a mindfulness practice. Psychoeducation will be delivered in written and animated video formats. A sample of these materials can be viewed in Multimedia Appendix 1. Each lesson will end with a reflection prompt that participants are encouraged to respond to in a journal to help generalize each lesson and apply them to their daily lives.

#### Mindfulness Practices

Beginning on day 2 of the intervention, participants will also receive a link to an audio file of a guided mindfulness practice via an automated text message each day. These mindfulness audio files will be hosted on SoundCloud, and a link will be texted to participants every morning. Mindfulness practices will range in duration and focus in an effort to build intensity slowly over the course of the intervention. For example, day 2 will include a 4-minute mindfulness of breath practice to complete after reading psychoeducation materials. Participants will be instructed to use the brief mindfulness practices during week 1 to get acquainted with what it feels like to be engaged in mindfulness, troubleshoot logistical barriers (eg, finding the ideal location and time of day), and identify additional barriers that may arise during their participation in Ruby (eg, noticing more unwanted unpleasant emotions) while engaging in supportive psychoeducation. As participants progress, mindfulness practices will begin to focus on self-compassion and finally, self-compassion as applied to body concerns. These practices are publicly available mindfulness practices written and recorded by Brach [22] and Neff [23]. Several of these practices were previously used in other investigations of compassion and body image concerns [18].

#### Daily Prompts

Daily tasks through days 1 to 28 will be automatically texted to every participant at 7:15 AM each morning. This will include links to psychoeducation materials and the accompanying animated video and mindfulness practice. Because of technical constraints, it is necessary for all participants to receive their daily prompts at the same time rather than allowing them to select the most convenient time for them. Participants will be instructed to complete their daily tasks or mindfulness practice at the most convenient time each day. There is little extant literature providing guidance on the preferred timing of text messages in clinical trials. There is some evidence that participants respond more favorably to text message prompts sent during waking hours rather than during sleeping hours [24]; thus, 7:15 AM was chosen as the ideal time as many people are awake and not yet at work at this hour. Participants will be encouraged to reach out to study staff if they would like assistance with troubleshooting the best time of day for completing their daily practice (eg, if they are shift workers or otherwise have an atypical schedule).

Participants will receive an additional text daily at 3 PM, which includes a reminder of their reflection for the day, a tip to troubleshoot mindfulness practice, a poem, or a note of compassion. In orientation materials, participants will be instructed to continue to follow the daily prompts even if they miss a day (rather than try to catch up on missed material). The additional texts (at 3 PM) will remind them of this instruction on an approximately weekly basis, and they will be reoriented to these instructions during their weekly self-monitoring feedback.

#### Weekly Self-monitoring

At the end of each week (ie, days 6, 13, 21, and 28), participants will be sent an automated text at 7 PM with a link to a weekly self-monitoring survey hosted on REDCap. Participants will report how many days that week they were able to complete their assigned tasks and will be provided automated feedback based on their responses.

If they practice for 0 to 3 days, they will provide feedback that includes encouraging messages and prompts to use self-compassion, identify specific barriers that got in the way of their practice, and create an action plan for how they could improve their adherence in the coming week. If they practice for 4 to 6 days, they will be provided with reinforcement and praise for completing their practice at least half of the week and will be asked to identify specific barriers and create an action plan for the coming week. If they practice every day, they will be provided with reinforcement and praise and will be asked to identify how they were able to practice every day and commit to doing the same in the coming week. If the participant does not respond to this survey, they will automatically be sent the survey again the following evening.

#### Compensation

Participants who complete all required study tasks (ie, informed consent, randomization, baseline surveys, and end-of-treatment surveys) will be entered into a raffle to win one of 20 Amazon e-gift cards valued at US $45 each. Participants will have a 25% chance of winning compensation. After completion of the study, 20 participant identifiers will be randomly selected using a random number generator. The participants will then have 72 hours to claim their compensation; if they are not responsive to emails in <72 hours, additional participant identifiers will be selected to maximize the number of participants who can receive compensation.

### Data Collection

Data collection for primary and secondary outcomes will be assessed at baseline (day 0) and end-of-treatment (day 28). All surveys will be administered via REDCap and are estimated to take 15-30 minutes to complete. A complete list of the surveys administered is presented in Table 1. Participants will be expected to complete their baseline assessments on the same day as they are deemed eligible. If a participant does not complete the baseline assessments that day, they will receive an automated email reminder that includes the link to the assessments. This reminder will be sent every 24 hours up to 3 times. If a participant does not complete the baseline assessments during the four-day window, they will be deemed no longer interested.

After completing the intervention, participants will receive additional text messages with instructions to complete the end-of-treatment surveys and receive a link to their surveys in their email. Participants will be encouraged to respond to the surveys within 24 hours. If a participant does not complete the surveys that day, they will receive an automated email reminder that includes the link to the surveys every 24 hours up to 5 more times. After 5 days of automated email attempts, study staff will personally reach out to participants via email to provide context about end-of-treatment surveys, remind them of the compensation they would be eligible to receive if they completed these surveys, and provide an opportunity for the participant to voice questions or concerns they may have about completing these surveys.

**Table 1:** Surveys administered at each timepoint.

Instrument	Time point					
	Screening	Day 0 (baseline)	Day 7	Day 14	Day 21	Day 28 (end-of-treatment)
Date of birth	✓^a^					
Anthropometric data	✓					
Modified weight bias internalization scale [5]	✓					
Demographics		✓				
Self-compassion scale [25]		✓				✓
Weight self-stigma questionnaire [26]		✓				✓
Fear of compassion scale [27]		✓				✓
Patient health questionnaire [28]		✓				✓
Weight and dieting history [29]		✓				
Intuitive eating scale [30]		✓				✓
International physical activity questionnaire (short form) [31]		✓				✓
Five facet mindfulness questionnaire (short form) [32]		✓				✓
Body appreciation scale [33]		✓				✓
Acceptance and action questionnaire for weight [34]		✓				✓
Childhood trauma questionnaire [35]		✓				
Weekly adherence survey			✓	✓	✓	✓
Acceptability and feasibility ratings						✓
Engagement and feedback report						✓

^a^Variable assessed.

### Measurements

#### Primary Outcomes

##### Weight Bias Internalization

Participants will complete the Modified Weight Bias Internalization Scale [5], a 10-item version of the prior Weight Bias Internalization Scale. This is a psychometrically validated assessment of weight bias internalization in people of all weight statuses, and the 10-item version of the original scale [36] was created to capture weight bias internalization regardless of whether the respondent was identified as overweight or obese [37]. Participants will be asked to rate their agreement with statements such as “My weight is a major way that I value myself as a person.” The Modified Weight Bias Internalization Scale is scored by computing the mean of 10 item responses rated on a 1 to 7 scale, with higher scores signifying greater weight bias internalization.

##### Weight Self-Stigma

Participants will complete the Weight Self-Stigma Questionnaire [26], a 12-item assessment of self-directed weight stigma. The Weight Self-Stigma Questionnaire captures different aspects of weight bias internalization and self-directed stigma compared with the Weight Bias Internalization Scale. Notably, in addition to the total score, the Weight Self-Stigma Questionnaire measures 2 distinct subscales: fear of enacted stigma and self-devaluation. Although both instruments are sound measures of weight bias internalization [38], they demonstrate different sensitivities to change in recent interventions of weight bias internalization [19]. Participants will be asked to rate their agreement with statements such as “I became overweight because I’m a weak person.”

##### Self-Compassion

Participants will complete the Self-Compassion Scale [25], a 26-item assessment of an individual’s capacity to direct compassion toward themselves. Participants will be asked how frequently they behave in a self-compassionate manner by responding to statements such as “I try to be loving toward myself when I am feeling emotional pain.” Per the recommendations by Neff [25], we will use the total score comprising all 6 factors in our primary outcome analysis. To understand the specific mechanisms that conferred changes in self-compassion global score, we will also analyze each of the 6 subfactors: the 3 compassionate self-responding factors (self-kindness, common humanity, and mindfulness) and the 3 uncompassionate self-responding factors (ie, self-judgment, isolation, and overidentification).

#### Secondary Outcomes

##### Mindfulness Constructs

The mindfulness constructs are as follows:

- Mindfulness: Participants will complete the Short Form Five Facet Mindfulness Questionnaire [32], a 24-item instrument that measures 5 facets of a tendency to be mindful in daily life. The 5 facets assessed are observing internal experience, describing internal experience, acting with awareness, nonjudging of inner experience, and nonreactivity to inner experience. Participants will rate their agreement with statements such as “When I have distressing thoughts or images, I don’t let myself be carried away by them.” Items are rated on a scale of 1 to 5, with higher scores indicating greater mindfulness.
- Weight-related experiential avoidance: Participants will complete the Acceptance and Action Questionnaire for Weight-Related Difficulties–Revised [34], a 10-item measure of experiential avoidance of unwanted thoughts, feelings, and actions related to weight. Data suggest that using a domain-specific inventory of experiential avoidance is more accurate than using the general Acceptance and Action Questionnaire. On this instrument, participants will be asked to rate their agreement with statements such as “When I evaluate my weight or appearance negatively, I am able to recognize that this is just a reaction, not an objective fact.” Items are rated from 1 (never true) to 7 (always true). Higher global scores reflect greater experiential avoidance. Three subfactors will also be analyzed: food as control (the tendency to use food as coping), weight as a barrier to living (tendency to move away from a valued life due to one’s body shape or weight), and internalized weight stigma.
- Fear of compassion: Participants will complete one subscale from the Fear of Compassion Scale [27]. The Fear of Compassion Scale measures compassion in the following three domains: compassion from others, compassion for others, and compassion for self. We will administer the 17-item fear of compassion for the self-subscale. Participants will rate their agreement with statements such as “I feel that I don’t deserve to be kind and forgiving to myself.” Items are rated from 0 (do not agree at all) to 4 (completely agree) and summed, with lower scores indicating lower fear of compassion.

##### Weight and Eating Constructs

The weight and eating constructs are as follows:

- Body appreciation: Participants will complete the Body Appreciation Scale 2 [33], a 10-item inventory assessing a sense of gratitude, appreciation, and positive attitudes toward the body. The original Body Appreciation Scale was updated to eliminate gendered language and update language, assuming that all respondents had
  body flaws
  that were inherently negative or looked upon unfavorably by the respondent. Participants will rate their agreement with statements such as “I appreciate the different and unique characteristics of my body.” Items are rated on a scale of 1 (never) to 5 (always); responses are averaged for a total score, with higher scores indicating higher body appreciation.
- Intuitive eating: Participants will complete the Intuitive Eating Scale-2 [30], a 23-item assessment of an individual’s tendency to eat in alignment with internal versus external cues. Participants will rate their agreement with statements such as “When I am craving a certain food, I allow myself to have it” on a scale of 1 (strongly disagree) to 5 (strongly agree), and a global score will be calculated, with higher scores indicating more intuitive eating patterns. We will also calculate each of the 4 subscales: unconditional permission to eat, eating for physical rather than emotional reasons, reliance on hunger and satiety cues, and body-food choice congruence.
- Weight and dieting history: Participants will respond to a series of questions selected from the Weight and Lifestyle Inventory [29]. We will select items that inquire about the frequency of dieting across their life span, age of first dieting experience, frequency of weight cycling episodes, history of eating disorder diagnosis or treatment, and specific dieting behaviors used (eg, caloric restriction and prescription medications). One weight cycle will be defined as a loss and regain of at least 10 pounds [39].
- Physical activity: Participants will complete the International Physical Activity Questionnaire–Short Form [31], a self-report inventory of recent physical activity across different domains (vigorous activity, moderate activity, walking, and leisure) in the past week.

##### Psychopathology and Trauma Constructs

The psychopathology and trauma constructs are as follows:

- Depression: Participants will complete the Patient Health Questionnaire 2 [28], a self-report instrument measuring depressive symptoms derived from the diagnostic criteria in the Diagnostic and Statistical Manual of Mental Disorders-IV. The 2-item version (abbreviated from the original 9-item Patient Health Questionnaire) does not include the suicidal ideation item and demonstrates good predictive validity with a cutoff score of ≥2 [40].
- Adverse childhood experiences: Participants will complete the Childhood Trauma Questionnaire [35], a 28-item retrospective assessment of childhood experiences of abuse, neglect, and maltreatment. The Childhood Trauma Questionnaire assesses experiences that are often not captured, such as chronic neglect, invalidation, psychological abuse, and other experiences that may not meet Diagnostic and Statistical Manual of Mental Disorders Criterion A trauma thresholds, yet significantly impact an individual’s capacity for healthy attachment, sense of self, and other measures of well-being. The Childhood Trauma Questionnaire has the following five subscales: emotional abuse, physical abuse, sexual abuse, emotional neglect, and physical neglect.
- Engagement and feedback: We will assess study adherence using weekly surveys in which participants will be asked to report the number of days they practiced mindfulness. Self-report adherence will also be assessed in the engagement and feedback surveys provided at study completion. In these surveys, we will ask participants to report what percentage of Ruby they believe they completed, which psychoeducation lessons and mindfulness practices they completed, and provide feedback on intervention content. We will also assess the usability and acceptability of Ruby at the end of the treatment.

### Analytic Approach

To describe baseline characteristics, we will compute descriptive statistics stratified by the treatment arm. To determine whether baseline characteristics differ by group assignment or retention status, we will use Pearson chi-square tests for categorical variables and analysis of variance for continuous variables.

We will use intent-to-treat analyses to test our primary study aim using one-way analysis of covariance models and linear mixed models to examine changes in weight bias internalization over time by treatment arm. Linear mixed models will be fitted with a full maximum likelihood estimation, and we will assume missingness at random. We will use an unstructured covariance matrix. We will not control for any additional variables. If the distribution of any outcome is heavily skewed, we will apply transformations to the data to conduct these analyses. We will assess the impact of moderating variables using analysis of covariance and linear mixed models. We hypothesize that the intervention effect may be moderated by certain demographic variables (ie, race or gender) or psychological variables (ie, fear of self-compassion or adverse childhood experiences determined by scores on the Childhood Trauma Questionnaire). We also hypothesize that the effect of the intervention will be partially mediated by an increase in self-compassion, measured using the Self-Compassion Scale. We will conduct mediation analyses using the SPSS PROCESS macro (IBM Corp) with 5000 bootstrap samples. Exploratory analyses will assess the moderating impact of additional constructs (eg, baseline depression scores) and the impact of the intervention on tertiary outcome variables (eg, body appreciation score and intuitive eating score). The sample size will likely not be large enough to be fully powered for these exploratory analyses, although they are worthy of investigation given the lack of clinical trials in this area. We will also conduct a per-protocol analysis with participants who report completing at least 60% of Ruby components for all analyses.

## Results

This protocol was designed in May 2020 and approved by the Duke University Institutional Review Board in December 2020. Data collection began in January 2021 and was completed in August 2021. Data analyses are underway, and the results are expected to be published in December 2021.

## Discussion

### Comparison With Prior Work

Ruby will be the first self-compassion-based intervention designed to reduce internalized weight bias. Although several cross-sectional investigations have indicated that self-compassion may be a construct of interest concerning internalized weight bias, Ruby is the first to test its efficacy in a randomized controlled trial. If efficacious, Ruby will be poised to expand a burgeoning body of literature related to psychological intervention in this area of need.

The nascent body of intervention research on internalized weight bias has demonstrated several promising results. Cognitive-behavioral strategies, such as the Weight Bias Internalization and Stigma (BIAS) program, have been tested to reduce internalized weight bias. The BIAS program has been tested in a pilot trial and a randomized controlled trial [19,41], testing an 8-week cognitive-behavioral intervention to reduce internalized weight bias compared with a quasi-control. The results of the pilot study suggest that the BIAS program can reduce weight bias internalization using cognitive-behavioral strategies. This program was then modified and tested in a randomized controlled trial with a longer duration (12 weeks) and combined with behavioral weight loss, compared with a behavioral weight loss program alone. No difference was observed between the 2 groups in the reduction of weight bias internalization when measured using the Weight Bias Internalization Scale, and some group differences were observed when weight bias internalization was measured using the Weight Self-Stigma Questionnaire. Overall, these results indicate that cognitive-behavioral interventions for internalized weight bias show some promise and require additional investigation. Further, differential results—depending on the self-report instrument used—raise important questions about measurements of this construct that should be explored in future work.

Acceptance- and mindfulness-based interventions for internalized weight bias show additional efficacy. Mindfulness strategies have been used several times to aid in obesity interventions, weight-related quality of life interventions, and recently, to improve body image concerns. Ruby is the first study to specifically test the efficacy of self-compassion mindfulness practices in reducing internalized weight bias. Albertson et al [18] demonstrated preliminary evidence that self-compassion could be useful in treating body image concerns, and the design of Ruby was inspired by their work, although there are some key differences. Albertson et al [18] trial was a 3-week intervention that provided self-compassion mindfulness audio files via podcast compared with a wait-list control. Ruby aims to provide additional psychoeducation about mindfulness, internalized weight bias, and the health effects of each.

Furthermore, the schedule of mindfulness practices differs when comparing Ruby with the Albertson et al [18] intervention; Ruby aims to build mindfulness skills slowly, beginning with brief practices (ie, 4 minutes) to improve accessibility for new meditators. Although both trials encourage daily mindfulness practice, Ruby’s mindfulness practices slowly increase in duration until they reach 20 minutes per day, whereas Albertson et al [18] opted to provide 20 minutes mindfulness recordings throughout the intervention. These key differences may or may not affect the efficacy of the intervention. Participants in the trial by Albertson et al [18] reported significant reduction in body dissatisfaction, reduction in body shame, and increase in body appreciation at 3 weeks. Moreover, these improvements remained 3 months after the completion of the study. Other studies have begun to explore the associations between acceptance-based strategies and body image concerns, such as the KgFree study [13].

### Strengths and Limitations

To date, most interventions for internalized weight bias have been lengthy and in-person interventions. Ruby may be an especially promising treatment for internalized weight bias for many reasons because of its remote, standalone digital delivery. First, remote, digital interventions can reach large portions of the population, given near-universal access to smartphones and other internet-capable devices across sociodemographically diverse communities. Even among individuals earning less than US $30,000 annually, more than 85% of US adults own smartphones, and rates of ownership increase among higher income levels [42]. This is particularly important because individuals living on lower incomes can have especially limited access to traditional in-person treatments for myriad reasons. Second, standalone digital interventions are more scalable than in-person or human-supported digital interventions, as they are not constrained by the availability of a finite number of health care providers.

Similarly, digital interventions may be more accessible than in-person treatment, as participants can access a digital intervention at times of the day most convenient in their schedule and are not burdened by in-person time commitments, which can be a barrier for participants with shift jobs, caregiver responsibilities, lack of transportation, and so on. Finally, the standalone digital delivery approach allows Ruby to operate at a much lower cost than human-supported treatments for weight bias. Although a standalone remote intervention may not be sufficient for all individuals, Ruby’s comparatively low resource footprint may make it a viable first option in a stepped care model. Remote, standalone digital interventions may be a particularly promising first-step intervention given the negative impact of internalized weight bias on help-seeking [4] and may be a useful alternative to group-based treatment that may prime individuals for comparison with others.

Owing to the brief, digital, standalone design of this trial, we believe Ruby could be easily disseminated and integrated into other evidence-based care packages to enhance their effects. Self-compassion training would make an excellent adjunctive treatment in populations that tend to have higher body weights, such as for interventions concerning physical activity, diabetes management, hypertension, or weight loss. Future investigations should explore the efficacy of Ruby in tandem with such treatments. If efficacious, Ruby may be able to improve treatment engagement and reduce downstream markers of chronic stress in these populations and improve overall treatment outcomes.

Recent popular media outlets have suggested that weight loss medications, such as semaglutide, may also result in reductions in weight stigma. Although any reduction in weight stigma is welcomed, we posit that reductions in weight stigma need not be tied to weight loss. It is well known that weight-related reductions in weight stigma in some populations are often reversed when weight is regained; thus, these reductions may be temporary and may send a message that patients must be thin to be free of weight stigma. Furthermore, investigations of stigma following bariatric surgery suggest that many patients experience lingering internalized weight bias even after losing significant amounts of weight because of the lasting effects of chronic stress and discrimination based on their weight. Finally, weight loss is not mandatory. Many Americans live long, healthy, and values-consistent lives at higher weights and should have access to low-cost, effective treatments to reduce internalized weight bias without inducing weight loss. For these individuals, Ruby may hold a promise.

## Appendix

Multimedia Appendix 1 Sample psychoeducation video delivered on day 1.

## Abbreviations

BIAS: Weight Bias Internalization and Stigma
REDCap: Research Electronic Data Capture
